# Fasting Enhances the Response of Glioma to Chemo- and Radiotherapy

**DOI:** 10.1371/journal.pone.0044603

**Published:** 2012-09-11

**Authors:** Fernando Safdie, Sebastian Brandhorst, Min Wei, Weijun Wang, Changhan Lee, Saewon Hwang, Peter S. Conti, Thomas C. Chen, Valter D. Longo

**Affiliations:** 1 Longevity Institute and Department of Biological Sciences, School of Gerontology, University of Southern California, Los Angeles, California, United States of America; 2 USC/Norris Neuro-Oncology Program, Neurological Surgery and Pathology, USC Keck School of Medicine, Los Angeles, California, United States of America; 3 Department of Radiology, Molecular Imaging Center, USC Keck School of Medicine, Los Angeles, California, United States of America; 4 Centre for Medical Biotechnology, Faculty of Biology, University Duisburg–Essen, Essen, Germany; Columbia University, United States of America

## Abstract

**Background:**

Glioma, including anaplastic astrocytoma and glioblastoma multiforme (GBM) are among the most commonly diagnosed malignant adult brain tumors. GBM is a highly invasive and angiogenic tumor, resulting in a 12 to 15 months median survival. The treatment of GBM is multimodal and includes surgical resection, followed by adjuvant radio-and chemotherapy. We have previously reported that short-term starvation (STS) enhances the therapeutic index of chemo-treatments by differentially protecting normal cells against and/or sensitizing tumor cells to chemotoxicity.

**Methodology and Principal Findings:**

To test the effect of starvation on glioma cells *in vitro*, we treated primary mouse glia, murine GL26, rat C6 and human U251, LN229 and A172 glioma cells with Temozolomide in *ad lib* and STS mimicking conditions. *In vivo*, mice with subcutaneous or intracranial models of GL26 glioma were starved for 48 hours prior to radio- or chemotherapy and the effects on tumor progression and survival were measured. Starvation-mimicking conditions sensitized murine, rat and human glioma cells, but not primary mixed glia, to chemotherapy. *In vivo*, starvation for 48 hours, which causes a significant reduction in blood glucose and circulating insulin-like growth factor 1 (IGF-1) levels, sensitized both subcutaneous and intracranial glioma models to radio-and chemotherapy.

**Conclusion:**

Starvation-induced cancer sensitization to radio- or chemotherapy leads to extended survival in the *in vivo* glioma models tested. These results indicate that fasting and fasting-mimicking interventions could enhance the efficacy of existing cancer treatments against aggressive glioma in patients.

## Introduction

Malignant glioma, including anaplastic astrocytoma and glioblastoma multiforme (GBM), account for more than 50% of all primary brain tumors, with GBM being the most common malignant brain tumor in adults [1]. GBM is highly invasive and angiogenic [2], resulting in mortality rates higher than those for any other brain tumor, with a median survival of 12 to 15 months [3], [4]. The multimodal treatment of GBM includes maximal surgical resection, followed by adjuvant radio- and chemotherapy [5], [6], [7]. Other than the introduction of the chemotherapeutic drug Temozolomide (TMZ) the treatment protocol has generally not changed over the past decades [2]. Because it is well tolerated and has been shown to prolong patient survival, TMZ is currently the standard chemotherapy drug adopted for the treatment of high grade glioma of astroglial origin [8], [9]. TMZ is a lipophilic prodrug that is converted to the active metabolite methyltriazenolimidazole-carboxamide at physiological pH [10], resulting in the formation of methyl adducts at the O^6^ position of guanine in the DNA [11]. This methylation leads to mismatch pairing with thymine during DNA replication and to subsequent DNA strand breaks which eventually cause apoptosis [12]. In GBM samples, TMZ resistance has been linked to the cellular expression of O^6^-methylguanine DNA methyltransferase (MGMT). MGMT actively repairs the DNA damage induced by TMZ treatment by removing the O^6^-methyl adducts [13]. In fact, the greatest survival benefit provided by TMZ treatment was reported for tumors containing a methylated MGMT gene, which has a reduced expression and activity of this repair protein [14], [15]. Despite the benefits of TMZ treatment, a cure for GBM remains elusive; and almost all patients suffer recurrence, underlining the importance of augmenting the efficacy of existing treatments as well as developing new therapeutics.

Most chemotherapeutic agents cause damage to normal cells and tissues, particularly to those with high proliferative indices such as the bone marrow, lung and gut [16], resulting in severe short- and long-term side effects which make toxicity the dose-limiting factor for most chemotherapeutic-treatments. We have previously demonstrated that fasting or short-term starvation (STS) can selectively protect normal cells, mice and potentially patients from chemo-toxicity without reducing the therapeutic effect on cancer cells [17], [18], [19], a phenomenon we termed Differential Stress Resistance (DSR). The starvation-induced DSR may be attributed to the redistribution of finite energy and resources from reproduction/growth to cellular protection/maintenance in normal, but not cancer cells [17], [20]. The coordinated physiological responses to nutrient scarcity are in part mediated by reduced insulin-like growth factor 1 (IGF-1) signaling and the subsequent activation of cellular protection mechanisms [19], [21]. In contrast, tumor cells harbor oncogenic mutation(s) in growth signaling genes, including IGF1R, PI3K, PTEN and Ras, which render them self-sufficient in proliferation signaling and unresponsive to starvation conditions [22]. Glucose is the main energy source for cells, particularly for highly proliferative ones such as malignant cells. Many cancer cells, including glioma cells, display elevated glucose uptake and glycolysis even in the presence of oxygen, a phenomenon known as the Warburg effect [23], [24], [25], [26], [27]. In fact, elevated blood glucose is associated with an increased cancer rate and is thought to be a major risk factor for a variety of malignancies [28], [29], [30]. We and others have previously demonstrated that reducing glucose and IGF-1, or inhibiting the downstream signaling of mTOR/S6K, elicit differential stress responses in normal-and cancer cells to chemotherapy drugs *in vitro* [17], [31]; and that short-term starvation can protect mice and potentially humans from the side-effects of high-dose chemotherapy [18], [19]. In addition to the fact that malignant cells are unresponsive to starvation-induced cellular protection, our recent study has shown that 15 out of the 17 murine and human cancer cell lines tested were sensitized to chemotherapeutic drugs Doxorubicin (DXR) and/or Cyclophosphamide (CP) under starvation mimicking conditions *in vitro*. In mice, STS in combination with DXR or CP resulted in enhanced treatment efficacy of a variety of malignancies, including murine breast cancer and melanoma, as well as human neuroblastoma, breast- and ovarian cancer [32].

Here we tested the hypothesis that short-term starvation can augment the efficacy of TMZ and radiotherapy, the standard treatments for glioma in the aggressive subcutaneous (s.c.) and intracranial (i.c.) murine models of GBM.

## Materials and Methods

### Cell Culture

The human glioblastoma cell lines U251, LN229 and A172 (ATCC) and the rodent GL26 (kindly provided by Dr. Linda Liau [33]) and C6 cells were maintained in DMEM (Invitrogen) supplemented with 10% fetal bovine serum (FBS) at 37°C under 5% CO_2_. Mouse primary mixed glia were obtained from the cerebral cortices of 1–3 day old C57BL/6 pups and cells were maintained in DMEM supplemented with 10% FBS.

### Treatments of Mammalian Cells

Cells were seeded into 96-well microtiter plates at 20,000 cells/well and incubated for 2 days in DMEM (4.5g/L glucose and 10% FBS) and washed with phosphate buffered saline (PBS) prior to treatments. Cells were then incubated in glucose free DMEM (Invitrogen) supplemented with 1% FBS and glucose (0.5 g/L,for starvation-mimicking condition, or 2.0 g/L, for *ad lib* condition) for 24 hours followed by the treatments with varying concentrations of Temozolomide (Temodar®, Schering, 2–8 mM) for 24 hours in DMEM modeling normal or starvation conditions. All treatments were performed at 37°C under 5% CO_2_. Cell survival was determined with the CytoTox 96® Non-Radioactive Cytotoxicity Assay (Promega) and presented as percentage of untreated control. Briefly, this assay quantitatively measures the release of lactate dehydrogenase (LDH) upon cell lysis. Released LDH in culture supernatants is measured using a coupled enzymatic assay, resulting in the reduction of a tetrazolium salt (INT) into a red formazan product.

### Short-term Starvation (STS)

All animal protocols were approved by the Institutional Animal Care and Use Committee (IACUC) of the University of Southern California. All mice were maintained in a pathogen-free environment throughout the experiments. 12-week old male C57BL/6N mice (Charles River) were used for all experiments. For short-term starvation (STS), mice were single caged and maintained in standard shoebox-cages without access to food for 48 hours. To avoid coprophagy or feeding on residual chow, cages were changed immediately before the initiation of STS. Animals had access to water at all times. Body weight of each individual animal was measured routinely during the STS regime to monitor the loss of body weight. Tumor inoculation, drug treatment, radiotherapy and tumor measurements were all performed under inhalant anesthesia (2% isoflurane).

### Subcutaneous Glioma Model

GL26cells in log phase growth were washed and suspended in PBS at 2×10^6^ cells/ml and injected subcutaneously (2×10^5^ cells/mouse in 100 µL PBS) in the lower back region of the mouse. Tumor size was measured routinely using a caliper. Tumor growth and survival data were plotted from pair matched, pooled experiments with the statistical software Prism (GraphPad Software).

### Intracranial Glioma Model

Mice were fixed in a stereotactic frame, a paramedian incision was made and a 1.5 mm bur hole was drilled in the right frontal lobe of the skull (1 mm anterior and 3 mm lateral relative to the bregma). Using a Hamilton syringe, 1×10^4^ GL26luc cells in 5 µL PBS were implanted 5 mm deep into the brain of each mouse. GL26luc cells are genetically engineered to express the firefly luciferase gene. Two animals received a mock surgery without the implantation of tumor cells. The skin incision was then closed with silk thread 3/0. To evaluate tumor progression, luciferin (50 mg/kg body weight) was administered via intra-peritoneal injections and animals were subjected to Bioluminescence Imaging (BLI) with a Xenon IVIS200 system at the USC Small Animal Imaging Center.

### Cancer Treatments

Temozolomide was delivered by injection through the lateral tail vein at 15 mg/kg body weight. To mimic multi-cycle treatments in humans, mice were injected in two rounds (at 24 and 48 hours of starvation for the STS+TMZ group) for a total of 30 mg/kg/cycle. Whole body irradiation was performed with Cesium 137 as radiation source at a dose of 5 Gy for the first treatment and 2.5 Gy for the second treatment, with or without 48 hour starvation. Mice were monitored daily. Animals showing signs of severe stress, deteriorating health status, or excess tumor load (s.c, 2500×mm^3^) were designated as moribund and euthanized. The data presented in the graphs showing moribund animals is based on these criteria.

## Results

### Glucose Restriction Sensitizes Glioma to Temozolomide Treatment in vitro

**Figure 1:**
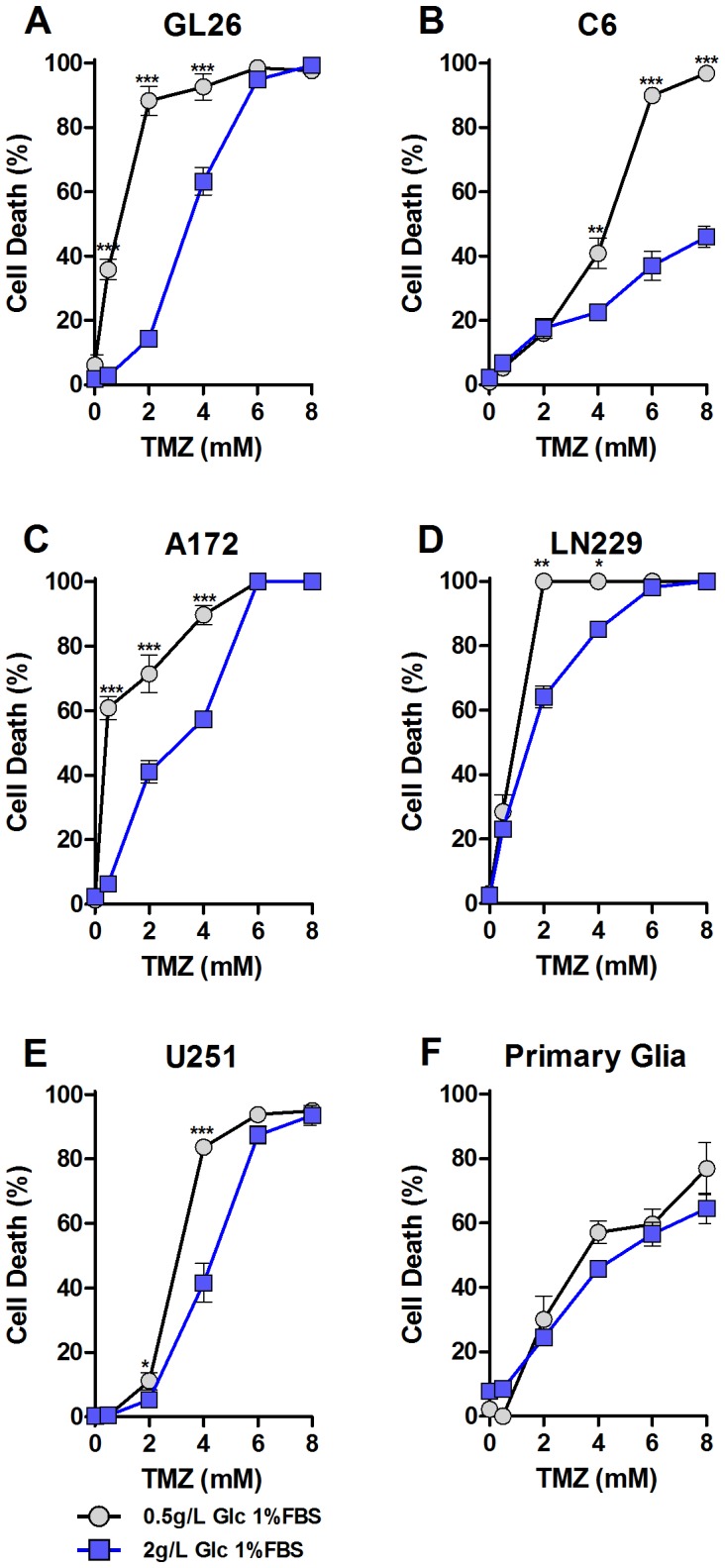
Glucose restriciton sensitizes Glioma Cells to Temozolomide Treatment in vitro. Glioma cell lines GL26, C6, LN229, A172 and U251 as well as murine primary mixed glial cell lines were tested for glucose-restriction induced sensitization to Temozolomide. Cells were incubated in low glucose (0.5 g/L) or normal glucose (2.0 g/L) media, supplemented with 1% FBS for 24 hours. Low glucose modeling STS conditions sensitized murine GL26 glioma cells (A), rat C6 glioma cells (B) and human A172 (C), LN229 (D), U251 glioma cells (E) to TMZ *in vitro*. (F) Murine primary mixed glial cells were used to represent matching normal cells. Percent cell death was determined based on quantitative measurements of lactate dehydrogenase (LDH) release after 24 hour treatment with 0–8 mM TMZ. All data presented as mean ± SEM; ** *p<*0.01; *** *p<*0.001, Student’s *t*-test, two-tailed.

We tested the effect of reduced glucose on cellular stress resistance to Temozolomide (TMZ), the standard chemotherapy drug for GBM treatment, *in vitro*. Cells were incubated in either low (0.5 g/L) or normal (2.0 g/L) glucose media for 24 hours prior to and during drug treatment to model the serum glucose levels during short-term starvation (STS) and *ad libitum* feeding, respectively. Murine GL26 cells maintained in medium mimicking blood glucose levels of starved mice displayed elevated cell death after TMZ treatment when compared to cells treated in normal conditions (Figure 1A). A 3-fold higher TMZ dose (6 mM vs. 2 mM) was necessary to cause ∼90% death in GL26 cells cultured in media containing 2.0 g/L glucose compared to 0.5 g/L glucose in the starvation-mimicking media (Figure 1A). Similar effects were seen with the human glioblastoma cell lines LN229, A172, and to a lesser extent, U251 cells, which displayed enhanced cytotoxicity at relatively low doses of TMZ treatment, suggesting that the sensitization of cancer cells to the TMZ treatment by glucose restriction may apply to human glioma (Figure 1B-E). In contrast to the malignant cells, TMZ toxicity to primary glia was not significantly affected by altering the glucose level (Figure 1F). These data suggest that glucose restriction sensitizes glioma cells, but not primary glia to TMZ treatment *in vitro*.

### Short-Term Starvation (STS) Improves the Efficacy of Chemo- and Radiotherapy in a Subcutaneous Glioma Model

**Figure 2:**
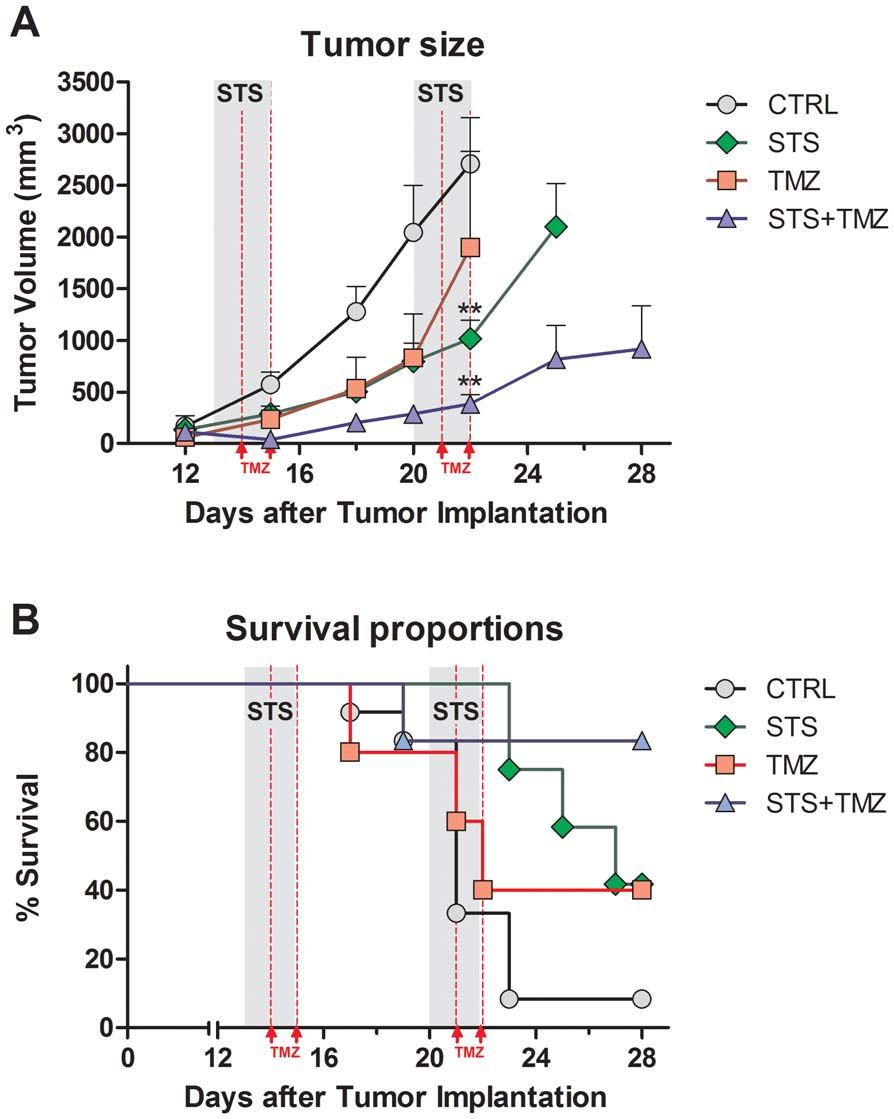
Enhanced Chemotherapy by Fasting in a murine GL26 Glioma Model extends onset of Morbidity in Tumor-bearing Animals. (A) Subcutaneous tumor progression of murine GL26 glioma is shown as total tumor volume in mm^3^. Tumor measurement was started once the tumor became palpable under skin at day 12. Control animals (N = 12) received no treatment and tumor progressed rapidly. STS (N = 12) and STS+TMZ group (N = 6) were deprived of food for two 48 hour cycles (day 13 to day 15 and day 20 to day 22, grey area). TMZ animals (N = 5) received 15 mg/kg/day TMZ (red lines), totalling 30 mg/kg for each treatment cycle. STS+TMZ animals were injected at 24 hours and 48 hours of fasting with 15 mg/kg TMZ per injection, totalling 30 mg/kg/cycle. Animals from STS and STS+TMZ groups showed reduced tumor progression and could therefore be monitored for a prolonged period compared to animals from the control and TMZ group. All data presented as mean ± SEM; ** *p<*0.01, ANOVA, Tukey’s multiple comparison, compared to control. (B) Morbidity of animals inoculated subcutaneous with GL26 glioma. Animals were euthanized once tumor volume exceeded 2500 mm^3^ or based on overall appearance and health status. Curve comparison with Log-Rank test (Mantel-Cox, *** *p<*0.05).

We evaluated the effects of combining STS and TMZ treatments in a subcutaneous (s.c.) model of GL26 glioma. Mice with tumor masses (s.c.) exceeding 2500×mm^3^ or showing signs of severe stress and deteriorating health (regardless of tumor load) were considered as moribund and sacrificed. GL26 tumors progressed rapidly in the untreated control animals, reaching the endpoint size of 2500×mm^3^ within 22 days after tumor implantation. Two cycles of treatment with TMZ (30 mg/kg each cycle) alone led to a deceleration of tumor progression, with the majority of the tumor-bearing animals in the TMZ treated group reaching the endpoint volume at day 22 and considerable variability (Figure 2A). Notably, two cycles of STS for 48 hours, in the absence of chemotherapy, retarded the progression of the glioma as effectively as the TMZ treatment during days 12 to 20 and slowed tumor growth even further during and after the second STS cycle in agreement with our recent results [32]. The greatest effect in decreasing tumor progression was observed when starvation was combined with the TMZ treatment for two consecutive treatment cycles (Figure 2A), which was associated with reduced serum glucose and IGF-1 levels (Figure S1).

Starvation alone did not cause obvious signs of discomfort, but instead improved the animal condition. In the STS+TMZ group, 85% of the mice appeared healthy with the tumor-size below the designated 2500 mm^3^ endpoint volume by day 28, indicating that the combination of both treatments was well tolerated and improved tumor-bearing survival (Figure 2B). In both the STS and TMZ groups, 40% of the animals survived until day 28 after tumor implantation. Notably, animals in the STS group had to be sacrificed solely based on tumor-size during days 23 to 28 while animals in the TMZ group showed an earlier onset of morbidity starting at day 17, in part caused by symptoms of chemo-toxicity, e.g. reduced food intake, loss of body weight and hypo-activity. On the other hand, survival rates were dramatically lower in the control group (8.3%). 50% of the untreated glioma-bearing mice reached the maximum tumor load by day 20; by day 22 all but one mouse in this group had to be sacrificed.

**Figure 3:**
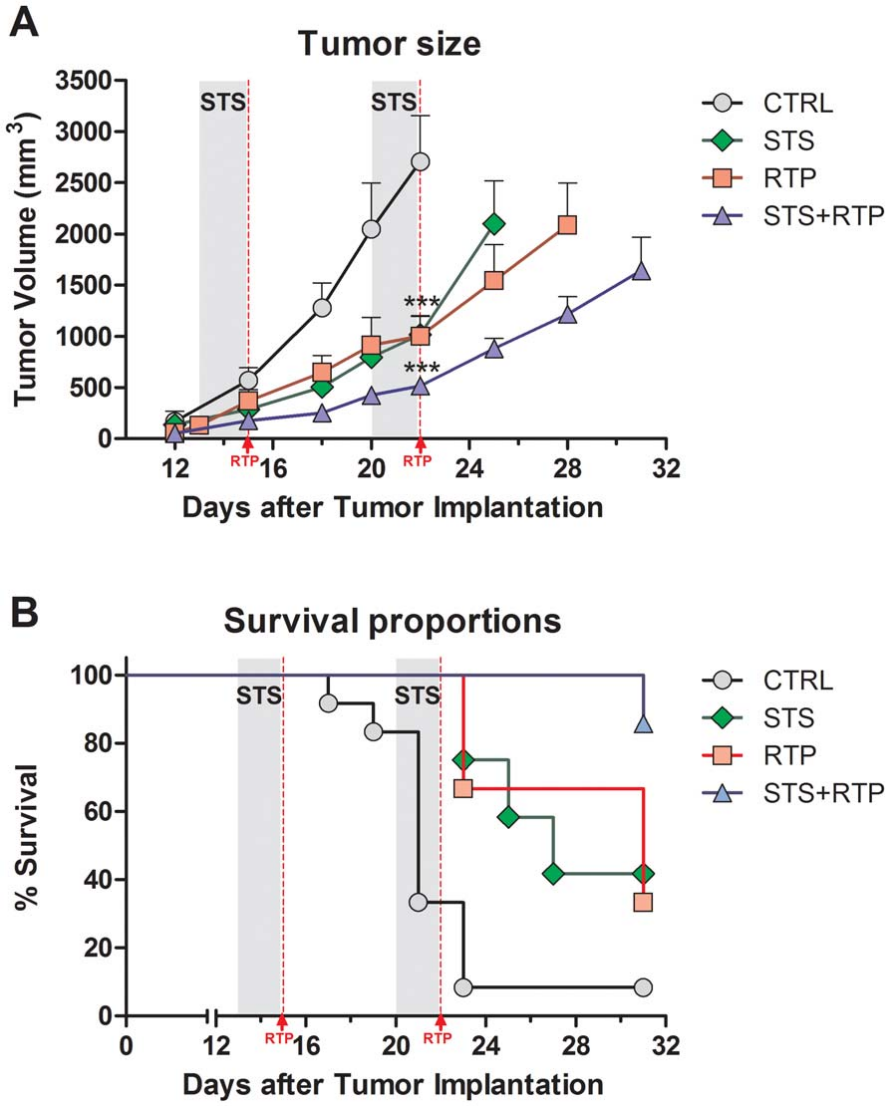
Enhanced Radiotherapy (RTP) by Fasting in a murine GL26 Glioma Model. (A) Subcutaneous tumor progression of murine GL26 glioma is shown by total tumor volume in mm^3^. Tumor measurement was started once the tumor became palpable under skin at day 12. Control animals (N = 12) received no treatment and tumor progressed rapidly. STS (N = 12) and STS+RTP group (N = 9) were deprived of food for two 48 hour cycles (day 13 to day 15 and day 20 to day 22, grey area). RTP and STS+RTP animals (N = 9) were treated with 5 Gy at day 15 and 2.5 Gy at day 22, totalling 7.5 Gy for the combined radiotherapy treatment; 2^nd^ dose was lowered to 2.5 Gy to reduce radiotoxicity. Animals from STS, RTP and STS+RTP groups showed reduced tumor progression and could therefore be monitored for a prolonged period compared to animals from the control group. All data presented as mean ± SEM; *** *p<*0.001, ANOVA, Tukey’s multiple comparison, compared to control at day 22. (B) Morbidity of animals inoculated subcutaneously with GL26 glioma and treated with either radiotherapy, two 48 hour fasting cycles or the combination of both (STS+RTP). Animals were considered moribund once tumor volume exceeded 2500 mm^3^ or based on overall appearance and health status. Curve comparison with Log-Rank test (Mantel-Cox; ** *p<*0.01).

Surgical resection followed by radiotherapy is another standard treatment for glioma patients. As STS delayed tumor progression and improved survival when combined with chemotherapy, we wanted to determine whether STS could also be beneficial in combination with radiotherapy. Radiotherapy (RTP) delivered in two cycles of 5 Gy and 2.5 Gy effectively retarded tumor growth when compared to the untreated control group (Figure 3A). Notably, up to day 22 the tumor progression for the RTP and STS groups was comparable. Once the second cycle of STS was stopped, a more rapid growth in the STS group was observed compared to that in the RTP group. The most striking effect was noticed in mice receiving two cycles of STS in combination with RTP, showing a significant reduction in tumor volume when compared to the control, STS and RTP groups.

As for the chemotherapy experiments, animals were considered moribund and were sacrificed once tumor volume reached 2500 mm^3^ or animals showed signs of a deteriorating health status (Figure 3B). Two cycles of radiotherapy alone were capable of significantly prolonging survival compared to the untreated control group; and ∼40% of the animals in this group survived until day 31, emphasizing the well-established benefits of radiotherapy for the treatment of GBM. As observed in the TMZ experiment, animals in the STS group displayed an enhanced survival when compared to the untreated control animals. Despite the fact that the tumor volume in the STS group progressed shortly after the 2^nd^ starvation cycle, animals in this group benefited with delayed tumor progression and showed extended survival. These data suggest that STS augments the efficacy of TMZ and RTP in treating aggressive GBM.

### Short-term Starvation Synergistically Improves the Efficacy of Chemotherapy in an Intracranial Model of Glioma

Malignant glioma account for approximately 70% of the 22,500 new cases of malignant primary brain tumors that are diagnosed in adults in the United States each year [34] with a 5 year survival rate that continues to be less than 3% [1]. Because of the high mortality rates and low median survival in brain tumor patients, we were interested in determining if the effect of fasting on an intracranial GBM tumor model would be as effective as that seen in the s.c. model. GL26 cells, engineered to express the firefly luciferase gene (GL26luc), were stereo-tactically implanted into C57BL/6 mice in the right frontal lobe, allowing us to monitor tumor progression by live bioluminescence imaging.

**Figure 4:**
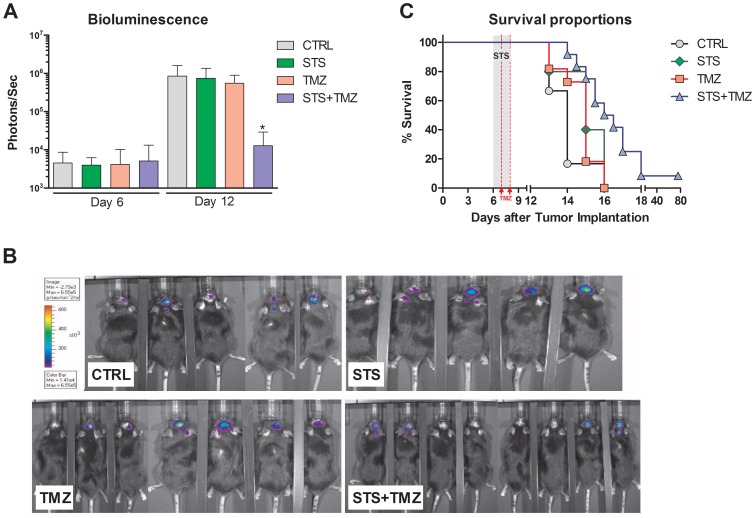
Fasting augments Effects of TMZ-Chemotherapy in the intracranial GL26luc Glioma Model. (A) Bioluminescence expression at day 6 vs. day 12 after tumor inoculation. Animals at day 6 were randomized into the four experimental groups (Control, N = 6; TMZ, N = 5; STS, N = 11; or STS+TMZ, N = 12) and treatment was initiated. Tumor progression was monitored at day 12 to determine treatment benefits. Bioluminescence signaling measured as photons/sec. All data presented as mean ± SEM; * *p<*0.05, ANOVA, Tukey’s multiple comparison, compared to control at day 12. (B) Bioluminescence imaging of GL26luc glioma bearing C57BL/6 mice at day 12 after intracranial tumor implantation. Animals are shown according to experimental group. (C) Morbididty rate comparison of animals inoculated intracranially with GL26luc glioma cells. STS and STS+TMZ animals were fasted for 48 hours starting at day 6 (grey area). TMZ and STS+TMZ animals received i.v. injections of 15 mg/kg Temozolomide at day 7 and day 8 (red lines), totalling 30 mg/kg/cycle. Curve comparison with Log-Rank test (Mantel-Cox; * *p<*0.05).

Within six days of tumor implantation, the luciferase expression by tumor cells could be measured in all mice by bioluminescence imaging (BLI, Figure S2). Based on the bioluminescence intensity, mice were randomized into four experimental groups to achieve an even distribution (Figure 4A, Day 6). TMZ treatment in human patients is performed as 5 consecutive daily injections per treatment cycle [8]. To accommodate the 48-hour STS regimen, TMZ was delivered by two injections of 15 mg/kg into the lateral tail-vein after 24 hours and 48 hours of starvation. On day 12 after the tumor implantation, the tumor progression in all experimental groups was assessed by BLI (Figure 4A, B). Bioluminescence in the control group increased within six days from ∼4500 to ∼850.000 photons/sec, an almost 200-fold increase. The STS and TMZ groups displayed a similar trend of increase in bioluminescence at day 12. However, one cycle of the combination of starvation and TMZ treatment reduced the bioluminescence signal of the implanted GL26luc cells significantly (*p<*0.05, compared to control at day 12).

The clinical condition and general health aspect of the mice declined rapidly, likely because of the increase in intracranial pressure caused by tumor proliferation. Amongst the most common manifestations of declining health was continuous weight loss in all groups, notable 6 to 8 days after the tumor implantation (Figure S3). Despite the weight loss during the starvation cycle, mice in the STS group regained the bodyweight to that of control animals within one to two days after resuming feeding. Furthermore, we also observed improved activity, exploring of the cage and better grooming in STS+TMZ treated mice when compared to animals from all other groups. These results are consistent with our previous observation that animals treated with chemotherapy after fasting display a better health status compared to their *ad lib* fed counterparts in chemotherapy-treated groups ([17], data not shown).

Mice that were starved prior to the administration of TMZ had a significant survival advantage (*p<*0.05) over the TMZ, STS and control groups in the intracranial GL26 tumor model (Figure 4C). Animals in the control, STS and TMZ groups showed an onset of morbidity 13 days after tumor implantation. The onset of morbidity caused by tumor burden was delayed by one day in the STS+TMZ group and survival was slightly prolonged in this group; consistent with the reduced bioluminescence signal at day 12. Remarkably, when considering the fast progression of this tumor model, one mouse from the STS+TMZ group was alive for more than 80 days after tumor implantation. These results suggest that fasting in combination with TMZ treatment can decelerate tumor progression in the mouse model and may potentially lead to long-term survival in a small sub-population.

**Figure 5:**
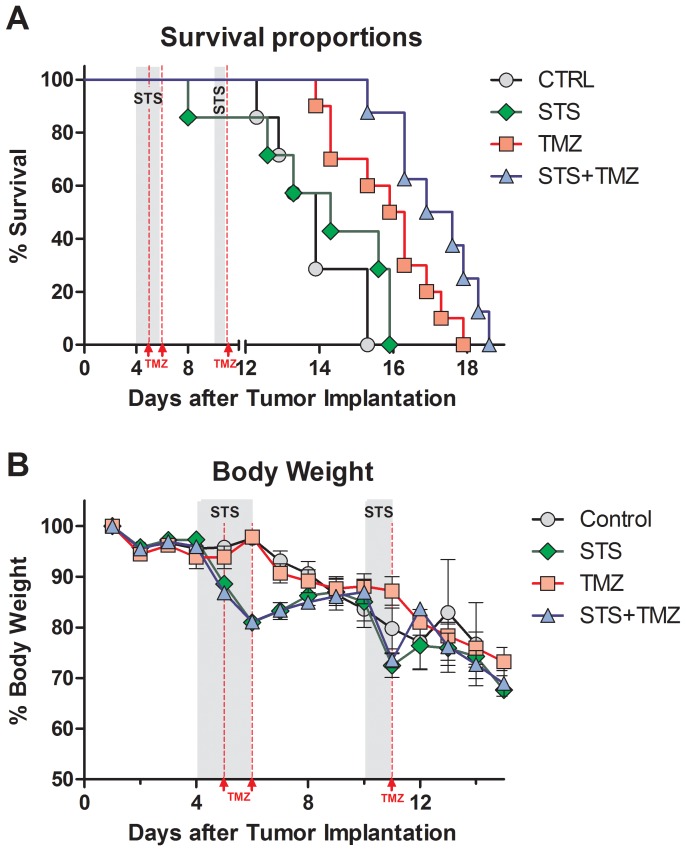
Two fasting cycles augment effects of TMZ-Chemotherapy in the intracranial GL26 Glioma Model. (A) Morbidity rate comparison of animals inoculated intracranially with GL26luc glioma cells. Animals at day 4 were randomized into the four experimental groups (Control, N = 7; TMZ, N = 7; STS, N = 10; or STS+TMZ, N = 8). Treatment of all experimental groups was initiated earlier to allow increased response to short-term starvation and/or TMZ. STS and STS+TMZ animals were fasted for 48 hours starting at day 4 followed by a second 24 hour fasting regiment at day 10 (grey areas). TMZ and STS+TMZ animals received i.p. injections of 15 mg/kg Temozolomide at day 5 and day 6 to match the first STS cycle and at day 11 to match the shorter second cycle (red lines), totalling 45 mg/kg. Curve comparison with Log-Rank test (Mantel-Cox; *** *p<*0.001). (B) Body weight profile for glioma-bearing animals receiving multiple rounds of either short-term starvation, TMZ-treatment or both. STS and STS+TMZ animals initially reduce body weight during STS cycles (grey area) but regain the weight of untreated control and TMZ-treated animals rapidly after refeeding.

To mimic oncologic treatment conditions in human patients, we attempted to extend survival by applying multiple cycles of chemotherapy treatment. As early as 4 days after implantation of the tumor, mice received two injections of TMZ (total 30 mg/kg, TMZ group) or were fasted for 48 hours (STS group). Mice in the STS+TMZ group received the combination of both treatments (Figure 5A). Before starting a second treatment cycle, we allowed animals from both STS groups to regain body weight to the values of non-starved groups (Figure 5B). By this time most animals had lost around 15% of bodyweight and appeared weak, making a shorter starvation period (24 hours) and a single dose of 15 mg/kg of TMZ at day 10 the only additional intervention possible. Two cycles of treatment (STS or TMZ) delayed the mortality of the tumor-bearing animals in comparison to the control group (Figure 5A), which showed a survival rate akin to that observed in our previous intracranial experiment (Figure 4C). Animals that received multiple doses of TMZ showed delayed onset of mortality compared to those in the control and STS groups (day 14 *vs.* day 12 and day 8, respectively). Furthermore, the onset of morbidity was delayed in comparison to the animals that received a single dose of TMZ in the previous experiment (Figure 4C, day 13). The additional 2^nd^ cycle of STS and TMZ further delayed morbidity in the experimental group receiving the combined treatments.

Despite our ability to delay the onset of mortality in tumor-bearing mice by intervening with treatments such as chemotherapeutic drugs and short-term starvation or the combination of both STS and TMZ, the extremely rapid progression of the intracranial GL26luc glioma model did not allow long-term survival. In a clinical scenario, where the tumor burden is reduced by resection or where the glioma is less aggressive, STS in conjunction with adjunctive therapy may offer potentially greater benefits.

## Discussion

The multimodal treatment of GBM, based on surgical removal of the tumor followed by chemotherapy and radiotherapy, has improved the survival of GBM patients. However, the frequency of recurrence and rapid progression in adults emphasizes the need for a major enhancement of the therapy to achieve long-term survival without relying exclusively on the uncertain and very long and expensive drug development process. In addition, chemotherapy often causes severe toxic side effects and might even fail due to the development of drug resistance as glioma, and GBM in particular, are among the most inherent chemotherapy- resistant tumors [35], [36]. The aim of this study was to investigate whether fasting, which could be rapidly, inexpensively and widely integrated into existing cancer treatments by clinicians, can improve the efficacy of chemo-and radiotherapies in treating mouse models of aggressive GBM. The intracranial inoculation of C57BL/6 mice with GL26luc cells showed a fast tumor progression that led to severe signs of illness such as back-hunching, reduced grooming (ruffled coat) and hypo-activity, possibly due to increasing intracranial pressure. One cycle of TMZ treatment at day 7 and 8 after tumor inoculation, or a 48 hour STS alone, lengthened the median survival from 14 to 15 days, although this effect was not statistically significant. By contrast, the combination of STS and TMZ delayed the onset of mortality but significantly extended median survival to 16 days. Remarkably, one animal in the STS+TMZ group achieved long-term survival. These results indicate that the combination of starvation with TMZ, the standard chemotherapy drug for the treatment of malignant glioma, has the potential to extend survival, at least in a portion of the subjects treated.

When we expanded the treatment to two cycles, we limited the 2^nd^ treatment to 24 hours of starvation because of a loss of body weight prior to STS, presumably due to tumor burden. Despite the shortened starvation regimen and the reduced TMZ dose, the STS+TMZ group showed an extended survival compared to that of the single cycle treated group (Figure 4, 5). Although this may be seen as a limited improvement for the treatment of advanced stage glioma, the fasting-based sensitization has the potential to achieve better health outcome in the treatment of a residual disease after the tumor resection.

The longer survival in the subcutaneous glioma model compared to the intracranial one allowed us to analyze the beneficial effects of STS in combination with TMZ-or radiotherapy. Both treatments, which are standard care in glioma treatment, significantly delayed tumor progression when combined with fasting. Notably, STS alone was capable of retarding tumor progression in the subcutaneous model to a degree comparable to that caused by radiotherapy during the earlier stages of tumor development. Thus, this dietary intervention may represent an alternative to patients who are unable to receive or opt out of the conventional treatments.

We have previously reported that a starvation-based approach was capable of differentially protecting normal cells and cancer cells against high-dose chemotherapy *in vitro* and showed improved survival and reduced tumor-size for neuroblastoma (NXS2) bearing animals treated with the chemotherapeutic drug etoposide *in vivo* [17]. Short-term starved mice were protected against 2–3 times higher doses of etoposide than the equivalent maximum human dose [17], [37]. Preliminary results from a case series study also suggest that fasting could protect patients against chemo-toxic side effects [18]. Thus, STS could allow the minimization of the systemic toxicity in the host while improving treatment outcome through higher doses and/or more frequent rounds of specific chemotherapies. Consistent with our STS-based approach, which results in blood glucose reduction, others have shown that the reduction in blood glucose availability, e.g. by 2-deoxy-D-glucose (a glucose analog and glycolytic inhibitor), can selectively enhance radiation-induced damage in tumor cells while protecting normal cells [38], [39]. However, fasting, which affects the levels of many growth factors and metabolites, is likely to be much more effective against a wider range of tumors than interventions that affect the levels of specific metabolites such as glucose.

Adaptation to fasting involves a multiplicity of physiological responses including metabolic changes, e.g. the switch to alternative nutrient sources due to reduced glucose availability and the reduction of growth factors such as IGF-1 [19], [40], [41]. Seyfried et al. demonstrated that calorie restriction (CR) reduced both biomarkers of tumor progression and angiogenesis [42], [43] and led to a reduction in tumor growth in a CT-2A malignant mouse astrocytoma model. However a 13 day 40% CR resulted in a 45% reduction in IGF-1, levels when compared to *ad lib* fed control animals, and also caused a major and chronic weight loss [44]. Short-term starvation reduces IGF-1 levels by 70%, reduces glucose by 60% (Figure S1B), increases IGFBP-1 11-fold and allows animals to regain their normal weight rapidly after re-feeding [19]. We have previously shown that STS can protect normal cells from and sensitize cancer cells to, both *in vitro* and *in vivo*, the cytotoxic effect of a range of chemotherapeutic drugs [17], [19], [32]. Our data here revealed that the beneficial effect of STS in cancer treatment is twofold: on the one hand, it dampens the levels of nutrients and growth factor signaling resulting in cellular protection of normal cells, but not cancer cells in which oncogenic mutations negate this adaptive stress resistance response; on the other hand, STS causes changes in the blood that promote cancer cell death and further sensitize cancer cells to cytotoxic stimuli, such as chemotherapy drugs and radiation. The contribution of the two aspects of STS-induced differential stress resistance, however, may be cancer cell type dependent. Our results here also indicate that reduced glucose levels are a significant component of the sensitization of GL26 glioma cells to TMZ *in vitro*. Furthermore, fasting also imparts a potent anti-inflammatory effect *in vivo* [45], whose role in enhancing cancer-therapy and reducing the side effects of the chemo-treatment warrants further investigation [46].

## Supporting Information

Figure S1 **Blood glucose and IGF-1 levels after 48 hour fasting in the murine GL26 glioma model (s.c.).** (A) Blood glucose levels of C57BL/6 mice after a 48 hour withdrawal of food (STS and STS+TMZ groups) or *ad libitum* fed control and TMZ treated animals were measured using a standard glucometer (ACCU-Chek). Blood was obtained by clipping the tip of the tail and glucose was read immediately. Control and TMZ animals were starved for 4 hours prior to blood withdrawal. (B) The effect of 48 hour fasting with TMZ treatment on serum IGF-1 was measured using IGF-1 specific ELISA (R&D Systems). Bar indicating median; ***p<0.001; ANOVA, Tukey’s multiple comparison, fasted vs. *ad li*b groups for glucose measurements; *t*-test, two-tailed for IGF-1.
(PDF)

Figure S2 **Bioluminescence imaging of intracranial GL26luc on day 6 after tumor inoculation.** Luminescent expression of intracranial GL26luc cells at day 6 after tumor inoculation was used to determine tumor-size. Animals were anesthetized using 2% isoflurane, injected with luciferin (i.p., 50 mg/kg body weight) and imaged using the Xenon IVIS system. Regions of interest (ROI) were used to obtain bioluminescence expression. Based on the signal strength, animals were assigned to experimental groups to achieve an even distribution prior to treatment initiation.
(PDF)

Figure S3 **Body weight profile for mice inoculated with GL26luc glioma intracranially.** Body weight profile for glioma-bearing animals receiving one cycle of either short-term starvation (STS), TMZ treatment or both. STS and STS+TMZ animals initially reduce body weight during STS cycles (grey area) but regain the weight of untreated control and TMZ-treated animals within 1 to 2 days after re-feeding. TMZ and STS+TMZ animals received i.v. injections of 15 mg/kg Temozolomide on day 7 and day 8 (red lines), totaling 30 mg/kg for the treatment cycle. Tumor progression causes rapid weight loss following treatment in all experimental groups while saline injected sham-animals (no tumor) recovered within one to three days from the inoculation procedure.
(PDF)
