# Supplementary figures and images for: Fasting Enhances the Response of Glioma to Chemo- and Radiotherapy
Source: PLoS One. 2012 Sep 11;7(9):e44603. doi: 10.1371/journal.pone.0044603 (PMC3439413; doi:10.1371/journal.pone.0044603)

**Figure S1**

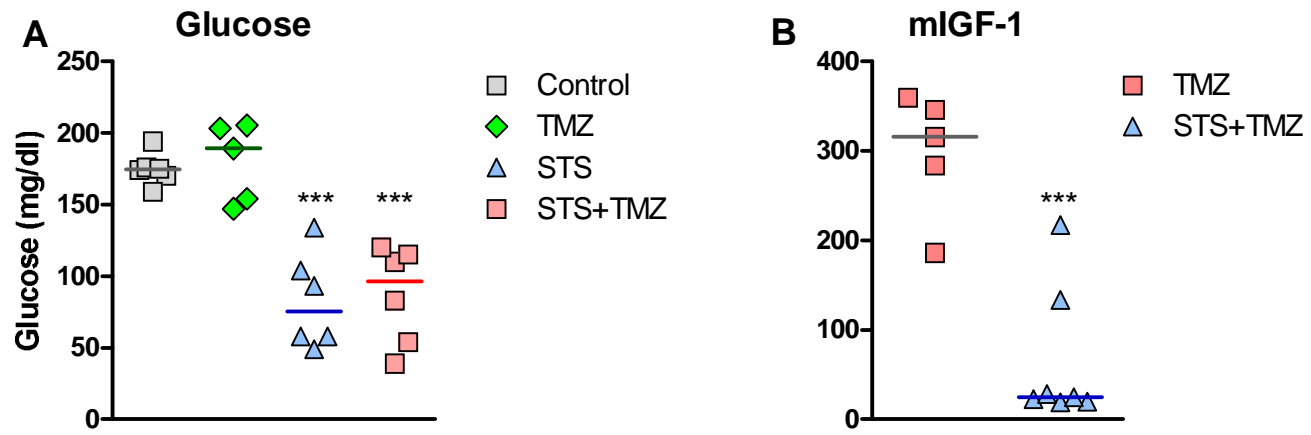

Supplement: Figure S1 — Blood glucose and IGF-1 levels after 48 hour fasting in the murine GL26 glioma model (s.c.). (A) Blood glucose levels of C57BL/6 mice after a 48 hour withdrawal of food (STS and STS+TMZ groups) or ad libitum fed control and TMZ treated animals were measured using a standard glucometer (ACCU-Chek). Blood was obtained by clipping the tip of the tail and glucose was read immediately. Control and TMZ animals were starved for 4 hours prior to blood withdrawal. (B) The effect of 48 hour fasting with TMZ treatment on serum IGF-1 was measured using IGF-1 specific ELISA (R&D Systems). Bar indicating median; ***p<0.001; ANOVA, Tukey’s multiple comparison, fasted vs. ad lib groups for glucose measurements; t-test, two-tailed for IGF-1. (PDF) [file pone.0044603.s001.pdf]

Figure S2

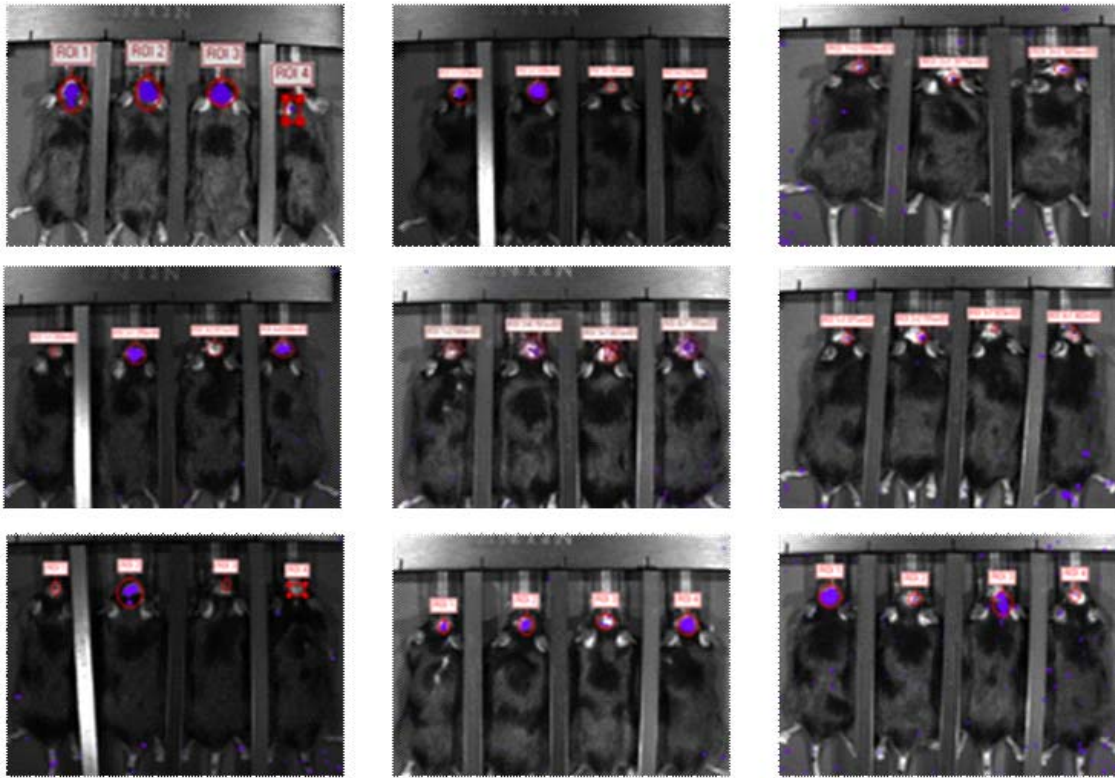

Supplement: Figure S2 — Bioluminescence imaging of intracranial GL26luc on day 6 after tumor inoculation. Luminescent expression of intracranial GL26luc cells at day 6 after tumor inoculation was used to determine tumor-size. Animals were anesthetized using 2% isoflurane, injected with luciferin (i.p., 50 mg/kg body weight) and imaged using the Xenon IVIS system. Regions of interest (ROI) were used to obtain bioluminescence expression. Based on the signal strength, animals were assigned to experimental groups to achieve an even distribution prior to treatment initiation. (PDF) [file pone.0044603.s002.pdf]

Figure S3

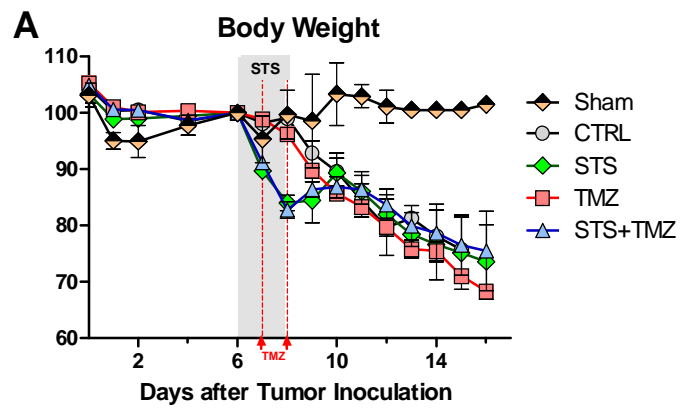

Supplement: Figure S3 — Body weight profile for mice inoculated with GL26luc glioma intracranially. Body weight profile for glioma-bearing animals receiving one cycle of either short-term starvation (STS), TMZ treatment or both. STS and STS+TMZ animals initially reduce body weight during STS cycles (grey area) but regain the weight of untreated control and TMZ-treated animals within 1 to 2 days after re-feeding. TMZ and STS+TMZ animals received i.v. injections of 15 mg/kg Temozolomide on day 7 and day 8 (red lines), totaling 30 mg/kg for the treatment cycle. Tumor progression causes rapid weight loss following treatment in all experimental groups while saline injected sham-animals (no tumor) recovered within one to three days from the inoculation procedure. (PDF) [file pone.0044603.s003.pdf]
